# The effect of interventions distributing home fortification products on infant and young child feeding (IYCF) practices: A systematic narrative review

**DOI:** 10.1111/mcn.13488

**Published:** 2023-02-26

**Authors:** Lindsey M. Locks, Katharine B. Newell, Annette Imohe, Grainne M. Moloney, Linda Shaker‐Berbari, Naveen Paudyal, Maria Elena D. Jefferds

**Affiliations:** ^1^ Department of Health Sciences, College of Health and Rehabilitation Sciences: Sargent College Boston University Boston Massachusetts USA; ^2^ Department of Global Health Boston University Boston Massachusetts USA; ^3^ United Nation Children's Fund (UNICEF) Headquarters New York City New York USA; ^4^ United Nation Children's Fund (UNICEF) Nepal Country Office Kathmandu Nepal; ^5^ Centers for Disease Control and Prevention Atlanta Georgia USA

**Keywords:** breastfeeding, complementary feeding, home fortification, infant and young child feeding, lipid‐based nutrient supplements, micronutrient powders, point‐of‐use fortification

## Abstract

Interventions distributing micronutrient powders (MNPs) and small‐quantity lipid‐based nutrient supplements (SQ‐LNS), or home fortification products (HFPs), have the potential to improve infant and young child feeding (IYCF) practices and children's nutrition. We systematically searched for studies on the effect of interventions distributing HFP on IYCF practices. We identified 12 (8 MNP, 4 SQ‐LNS) studies: seven programmes with IYCF behaviour change communications (BCC) and MNP (IYCF‐MNP) and one provided MNP without IYCF BCC (MNP only). Three SQ‐LNS studies came from randomised trials without an IYCF component (SQ‐LNS only) and one from a programme with both IYCF BCC and SfQ‐LNS (IYCF‐SQ‐LNS). Five IYCF‐MNP programmes reported positive associations with some IYCF practices—four with minimum dietary diversity, two with minimum meal frequency, four with minimum acceptable diet, and three with the initiation of complementary foods at 6 months. Two reported no association between MNP and IYCF indicators, and one reported a decline in IYCF practices during the intervention, although it also reported significant changes to the IYCF programme during the evaluation period. Two studies from interventions that distributed SQ‐LNS (one from a related set of randomised controlled trials and the sole IYCF‐SQ‐LNS programme) reported a positive association with IYCF practices; one trial reported no change in breast milk intake with the provision of SQ‐LNS and one found no association with IYCF practices. SQ‐LNS and MNP can address nutrient gaps for young children in low‐resource settings; our findings indicate that programmes that combine HFP with IYCF interventions may also contribute to improved IYCF practices in some settings.

## Key messages

- We identified 12 studies distributing home fortification products (HFP) that assessed infant & young child feeding (IYCF) outcomes; few used experimental or quasi‐experimental study designs.
- Interventions associated with improved IYCF practices were predominantly programs that integrated HFP with a new, intensive IYCF behavior change strategy.
- HFP can fill nutrient gaps for infants and children in low‐resource settings; programs that combine HFP with IYCF interventions may also contribute to improved IYCF practices in some settings.

## Graphical abstract

We systematically searched for studies on the effect of interventions distributing home fortification products (HFPs) on infant and young child feeding (IYCF) practices and identified 12 (8 micronutrient powders, 4 small‐quantity lipid‐based nutrient supplements) studies. Some interventions found an association with improved IYCF practices, and most of the interventions with significant associations were integrated programmes that included the distribution of HFP with a new, intensive IYCF behaviour change strategy that included frequent interpersonal counselling.

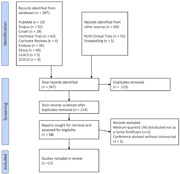

**Abbreviations**
AOR: adjusted odds ratio
APR: adjusted prevalence ratio
iLiNS: international lipid‐based nutrient supplements
ISSSF: introduction of solid, semisolid or soft foods
IYCF: infant and young child feeding
MAD: minimum acceptable diet
MDD: minimum dietary diversity
MMF: minimum meal frequency
MNP: micronutrient powders
RCT: randomised controlled trial
SQ‐LNS: small‐quantity lipid‐based nutrient supplements

## 1 INTRODUCTION

Malnutrition during early life increases the risk of mortality and morbidity in children and has lifelong consequences for physical and cognitive development (Black et al., 2013). Improving infant and young child feeding (IYCF) practices is essential to reduce stunting and other forms of malnutrition (Bégin & Aguayo, 2017). The World Health Organization (WHO) recommendations on IYCF include immediate and exclusive breastfeeding for the first 6 months, and the introduction of solid and semisolid complementary foods for children at 6 months of age when breast milk is no longer sufficient to meet children's nutritional needs (World Health Organization [WHO], 2003). In resource‐limited settings, nutrient‐rich foods are often inaccessible and/or are not provided in sufficient quantities to young children (Dewey et al., 2009). Fewer than one in five children aged 6–23 months in low‐ and middle‐income countries consumes the minimum acceptable diet (MAD), defined as a combination of an age‐based minimum meal frequency (MMF) of complementary foods and the consumption of minimum dietary diversity (MDD) (United Nations Children's Fund [UNICEF], 2020).

Home fortification (or point‐of‐use fortification) is an approach to improving the nutrient intake of young children and other nutritionally vulnerable groups which involves the addition of micronutrient powders (MNPs) or other specialised fortified products, such as lipid‐based nutrient supplements (LNS), to foods immediately before consumption (Dewey et al., 2009; World Health Organization [WHO], 2011). Common home fortification products include MNP and small‐quantity lipid‐based nutrient supplements (SQ‐LNS). MNPs are single‐dose, lightweight, shelf‐stable sachets designed to be mixed with a variety of semisolid foods to increase the availability of vitamins and minerals in children's diets (Zlotkin et al., 2005). SQ‐LNS are sachets that contain micronutrients as well as protein and essential fatty acids providing energy. SQ‐LNS typically contain ≤20 g (≤120 kcal or ~4 teaspoons) of an LNS, usually comprised of peanuts, vegetable oil, milk powder, sugar and added vitamins and minerals (Food and Nutrition Technical Assistance III Project [FANTA], 2016). In contrast to medium and large‐quantity LNS, which are primarily distributed as part of treatment for acute malnutrition, SQ‐LNS are usually provided for home fortification or for direct consumption to prevent undernutrition (Arimond et al., 2015).

Multiple meta‐analyses have demonstrated that MNP can reduce the prevalence of anaemia and iron deficiency (De‐Regil et al., 2013; Salam et al., 2013; Suchdev et al., 2020; World Health Organization [WHO], 2016) and that SQ‐LNS can reduce the risk of stunting, wasting and other forms of undernutrition in children aged 6–23 months (Das, Salam, Hadi, et al., 2019; Dewey, Stewart, et al., 2021; Dewey, Wessells, et al., 2021). The integration of these products into IYCF interventions may also influence IYCF practices (Siekmans et al., 2017). Unlike industrial fortification strategies (when nutrients are added during industrial food processing to household food items such as cooking oil or staple grains), home fortification allows for the targeting of young children with specific doses of several nutrients. Home fortification also, however, requires behaviour change on the part of the caregiver, who must regularly mix the fortification product into the child's food. It has been proposed that incorporating home fortification products into IYCF programmes could contribute to improved IYCF practices by establishing an enabling environment at the governmental and community levels to support IYCF programmes, and through the synergies created by providing a comprehensive package that includes counselling as well as improved access to essential nutrients (Siekmans et al., 2017). In programmatic settings, home fortification distribution is often implemented alongside enhanced IYCF trainings for facility‐based and community‐based health workers (CHWs) and behaviour change materials that re‐enforce key IYCF messages. It is also possible that the introduction of a tangible good (the fortification product) for CHWs to distribute in the community, may improve the demand for and delivery of CHW services (Locks et al., 2018; Reerink et al., 2017; Vossenaar et al., 2017), which could, in turn, improve IYCF practices as well as other child health and nutrition outcomes. Conversely, there is also concern that home fortification products could negatively impact IYCF by becoming a distraction for caregivers and/or by directly displacing breast milk or other foods, particularly in the case of LNS or other fortification products that include macronutrients (Flax et al., 2015; Kumwenda et al., 2014). To minimise disruption of IYCF practices, it is frequently recommended that SQ‐LNS is mixed into local complementary foods (as opposed to direct consumption) (FANTA, 2016). To determine whether interventions that distribute home fortification products influence IYCF practices, we conducted a systematic search for studies from interventions that provide these products and reported on the effect on IYCF practices.

## 2 METHODS

**Figure 1:**
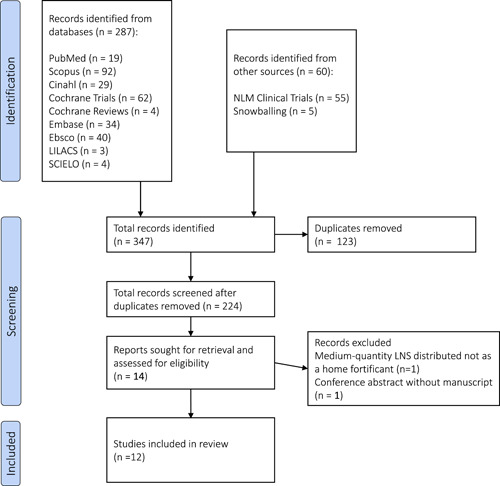
PRISMA diagram. LNS, lipid‐based nutrient supplements.

A systematic search in accordance with the PRISMA guidelines (Page et al., 2021) was conducted by a CDC staff librarian (Figure 1). First, they searched PubMed, Embase, Scopus, Ebsco Academic Search Complete, Cochrane Reviews, LLILACS and SCIELO for the following keywords in the title or abstract, or used relevant Mesh terms in PubMed: ‘infant or child with micronutrient powders’, ‘small quantity lipid‐based nutrient supplements’, ‘SQ‐LNS’, ‘fortification or fortificant’. The search led to 224 independent articles after de‐duplification. Two independent readers then screened the abstracts and titles of all of the articles with the following inclusion criteria: (1) English or Spanish language; (2) an intervention that provided home fortification products to children aged under 24 months—intervention was broadly defined to include trials, programmes, policies; home fortification products was broadly defined as any product intended to be added to foods to increase the quantity of nutrients or bioavailability of nutrients to prevent malnutrition; and (3) reported on IYCF outcomes—broadly defined to include any indicators related to breastfeeding and complementary feeding practices (including dietary diversity and frequency). We excluded articles that did not explicitly use a home fortification product for the prevention of malnutrition (i.e., medium or large‐quantity LNS as part of treatment for malnutrition) and also excluded articles that only reported on the effect of home fortification products on biological outcomes (i.e., anthropometry and anaemia) without including IYCF indicators. Fourteen articles fulfilled the inclusion criteria and their full texts were retrieved for further review of the full article to further determine eligibility for inclusion. We ultimately excluded one abstract from a conference that did not have a full‐text manuscript (Matias & Vargas‐Vasquez, 2017) and one trial that provided a larger LNS quantity (46.3–70 g) and did not explicitly counsel caregivers to mix the LNS with food (Flax et al., 2015).

## 3 RESULTS

Our final review included 12 articles—eight on MNP interventions (Ford et al., 2021; Geletu et al., 2019; Lanou et al., 2019; Locks et al., 2017, 2018; Mirkovic et al., 2016; Tariqujjaman et al., 2021; Young et al., 2021) and four on SQ‐LNS interventions (Arimond et al., 2015; Byrd et al., 2019; Kumwenda et al., 2014; Locks, Nanama, et al., 2019); no other home fortification products were discovered during our search. Table 1 shows all studies from interventions distributing MNP were from integrated IYCF‐MNP programmes or pilot programmes that provided MNP along with nutrition education and/or counselling on IYCF except one study from a pilot MNP programme that did not include an IYCF behaviour change component (Geletu et al., 2019). Among the four papers reporting on the effect of an SQ‐LNS intervention on IYCF practices, three of the articles report results from randomised controlled trials (RCTs) and only one from a programmatic setting (Table 2). Two RCT studies included nutrition or IYCF messages, one RCT with eight sites included monthly home visits delivering IYCF counselling and free SQ‐LNS in the two ‘nutrition’ arms, and one pilot programme included IYCF counselling.

**Table 1:** Studies assessing the effect of interventions with micronutrient powders (MNP) on Infant and young child (IYCF) practices.

Author (year)	Location	Intervention	Study design	Findings	Association w/IYCF indicators
Ford et al. (2021)	*Uganda*	*Pilot IYCF‐MNP Program*	*Quasi‐Experimental* *n* = 2816 Pre and postintervention surveys in representative samples of intervention and control districts Intervention: MNP + IYCF BCC Control: standard of care	*Intervention positively associated with MMF and age at introduction of complementary foods*	*Positive* *(MMF & age at introduction of complementary foods)* *Null* *(MDD & MAD)*
				Adjusted prevalence DiD analyses (95% CI), *p* value MDD: +6.0% (−2.2, 14.1), *p* = 0.10; Prevalence at baseline → endline Intervention: 18.3% → 59.9% Control: 26.6% → 61.6% MMF: +18.6% (11.2, 25.9), *p* < 0.0001; Intervention: 16.4% → 27.1% Control: 33.8% → 25.3% MAD: +5.6% (0.02, 11.2), *p* = 0.05 Intervention: 4.8% → 19.1% Control: 10.7% → 18.9% Introduction of complementary foods at 6 months: +21.7% (13.4, 30.1) *p* < 0.0001 Intervention: 42.0% → 66.6% Control: 57.1% → 59.3%	
Geletu et al. (2019)	*Ethiopia*	*Pilot MNP program* No IYCF component	*Observational* *n* = 200 Postintervention survey in intervention area only; sampling based on MNP exposure; primary aims were to compare anaemia, stunting and ECD Intervention: District‐wide MNP distribution Control: Individual children retroactively identified as ‘exposed’ or ‘unexposed’ to MNP	*No association between MNP exposure and IYCF practices*	*Null*
				Exposed vs. Unexposed prevalence, *p* value from Mann–Whitney *U* test. MDD: 3.1% vs. 3.9%, *p* = 0.74 MMF: 73.5% vs. 73.5%, *p* = 0.99 MAD: not reported Timing of introduction of solid food: not reported	
Lanou et al. (2019)	*Burkina Faso*	*Pilot IYCF‐MNP Program*	*Experimental* 62 clusters, *n* = 2396 Cluster RCT with pre and postintervention surveys in intervention and control clusters (villages) Intervention: MNP + IYCF BCC Control: standard of care (growth monitoring & promotion with referral of MAM/SAM cases)	*Intervention positively associated with MDD, MAD and age at introduction of complementary foods*.	*Positive* *(MDD, MAD & timely introduction of complementary foods)* *Null* *(MMF)*
				Adjusted prevalence DiD (95% CI), *p* value:	
				MDD: +6.5% (0.2, 12.7), *p* = 0.04; Prevalence at baseline → endline Intervention: 8.2% → 8.4% Control: 11.8% → 5.5% MMF: +5.8% (−13.3, 24.8), *p* = 0.55; Intervention: 80.6% → 91.8% Control: 71.4% → 76.6% MAD: +5.8% (0.2, 11.9), *p* = 0.04 Intervention: 7.6% → 7.9% Control: 10.9% → 5.2% Consumption of solids in children 6–8 months (*n* = 494): +17.9% (1.5, 34.2), *p* = 0.03 Intervention: 29.5% → 34.5% Control: 31.3% → 18.5%	
Locks et al. (2017)	*Madagascar*	*Pilot IYCF‐MNP Program*	*Observational* *n* = 847 Pre and postintervention surveys in representative sample in intervention district Intervention: MNP + IYCF BCC Baseline (no control group): standard of care	*Intervention positively associated with MDD and MAD*.	*Positive* *(MDD, MAD)* *Null* *(MMF & timely introduction of complementary foods)*
				Prevalence ratios compare endline (April 2014) to baseline (Oct/Nov 2012). APR (95% CI), *p* value: MDD: 2.92 (2.24, 3.80), *p* < 0.001 Baseline → Endline: 14.9% → 48.0% MMF: 0.95 (0.88, 1.03), *p* = 0.24 Baseline → Endline: 79.7% → 77.8% MAD: 2.88 (2.17, 3.82), *p* < 0.001 Baseline → Endline: 14.0% → 44.8% Consumption of solids in children 6‐8 months (*n* = 144): 0.99 (0.86, 1.14), *p* = 0.86 Baseline → Endline: 83.9% → 84.7%	
Locks et al. (2018)	*Nepal*	*IYCF‐MNP program* (at scale)	*Observational* *n* = 5121 Pre and Postintervention surveys in representative sample in 2 intervention districts Intervention: MNP + IYCF BCC (individual & group counselling by CHWs and health facility staff, mass media & IEC) Baseline (no control group): standard of care	*Intervention positively associated with MDD, MAD and age at introduction of complementary foods*.	*Positive* *(MDD, MAD, age at introduction of complementary foods)* *Null* *(MMF)*
				Adjusted prevalence ratios compare endline (Jan–Feb 2016) to baseline (Feb 2013)—APR (95% CI), *p* value in Kapilvastu (K) and Achham (A) districts:	
				MDD: 1.48 (1.33, 1.66), *p* < 0.001 and 1.19 (1.06, 1.34), *p* = 0.004 K Baseline → Endline: 24.6% → 43.5% A Baseline → Endline: 26.7% → 36.5% MMF: 1.26 (1.13, 1.40), *p* < 0.001 and 1.02 (0.95, 1.09), *p* = 0.62 K Baseline → Endline: 45.0% → 55.8% A Baseline → Endline: 57.5% → 56.2% MAD: 1.77 (1.52, 2.07), *p* < 0.001 and 1.19 (1.02, 1.39), *p* = 0.02 K Baseline → Endline: 14.5% → 30.1% A Baseline → Endline: 18.5% → 24.6% Introduction of complementary foods at 6 months: 1.68 (1.53, 1.84) *p* < 0.001 and 1.67 (1.54, 1.82) *p* < 0.001 K Baseline → Endline: 35.9% → 64.7% A Baseline → Endline: 48.6% → 82.9%	
Mirkovic et al. (2016)	*Nepal*	*Pilot IYCF‐MNP Program*	*Observational* *n* = 1708 Postintervention survey in representative sample in intervention areas only (at 3 months in 2 districts & 15 months in 2 districts); Intervention: MNP + IYCF BCC Baseline (no control group): standard of care	*Effective coverage positively associated with MDD, MMF and MAD*.	*Positive* *(MDD, MMF & MAD)*
				Adjusted odds ratios (AORs) comparing children who consumed 30–60 MNP sachets vs. none at endline in two surveys conducted after 3 months and 15 months, respectively:	
				MDD: 2.56 (1.80, 3.64) and 2.22 (1.55, 3.19) 3‐months high‐consumers vs. no MNP: 47.0% vs. 26.6% 15‐months high‐consumers vs. no MNP: 58.1% vs. 30.1% MMF: 2.04 (1.40, 2.97) and 1.38 (0.95, 2.01) 3‐months high‐consumers vs. no MNP: 72.8% vs. 57.8% 15‐months high‐consumers vs. no MNP: 77.4% vs. 68.3% MAD: 2.52 (1.68, 3.78) and 2.27 (1.59, 3.23) 3‐months high‐consumers vs. no MNP: 36.7% vs. 19.5% 15‐months high‐consumers vs. no MNP: 48.6% vs. 22.4% Consumption of solids in children 6–8 months: insufficient power to compare high and low consumers	
Tariqujjaman et al. (2021)	*Bangladesh*	*IYCF‐MNP program* (at scale)	*Observational* *n* = 6479 Pre, Mid and PostIntervention surveys in representative samples in intervention areas only + qualitative research Intervention: MNP + IYCF BCC Baseline (no control group): IYCF BCC; change in IYCF program mid‐evaluation	*Intervention associated with decline in IYCF practices*.	*Negative*
				Three different survey groups: group #1: Sept 2014–2017; #2: Mar/Ap 2015–2018; #3: Ap/May 2016– 2018) Does not report MDD, MMF or MAD Prevalence of good IYCF practices (ICFI score > 6) at baseline, midline and endline were 42.1%, 45.3% and 31.9%, respectively.	
Young et al. (2021)	*India*	*Pilot IYCF‐MNP program*	*Experimental* Cluster RCT with pre and postintervention surveys in intervention and control clusters (70 health sub‐centers & >4000 children) Intervention: MNP + IYCF counselling Control: IYCF counselling only	*Intervention not associated with IYCF indicators*.	*Null*
				Adjusted prevalence DiD (95% CI); (*p* values not provided)	
				MDD: +0.2% (−4.8, 4.5) Prevalence at baseline (endline not provided) Intervention: 20.3% Control: 20.3% MMF: −0.6% (−5.3, 4.2) Intervention: 64.5% Control: 67.1% MAD: +1.1% (−2.9, 5.0) Intervention: 14.4% Control: 16.0% Initiation of solid foods between 6 and 7 month: +1.7 (−3.8, 7.1) Intervention: 37.8% Control: 39.3%	

Abbreviations: Ap, April; APR, adjusted prevalence ratio; BCC, behavior change communication; DiD, difference in difference; IYCF, infant and young child feeding; MAD, minimum acceptable diet; Mar, March; MDD, minimum dietary diversity; MMF, minimum meal frequency; MNP, micronutrient powder; RCT, randomized controlled trial; Sept, September.

**Table 2:** Studies on the effect of interventions with home fortification with small‐quantity lipid‐nutrient supplements (SQ‐LNS) on infant and young child feeding (IYCF) practices.

**Author (year)**	**Location**	**Intervention**	**Study design**	**Results**	**Association w/IYCF indicators**
Arimond et al. (2017)	*Burkina Faso, Ghana and Malawi (two trials)*	*Four related RCTs of SQ‐LNS*: RCTs tested different LNS regimens in women and children; no IYCF BCC component; but brief nutrition messages conveyed	*Experimental* *n* = 5658 Pooled data from four RCTs, two trial sites distributed LNS and two did not	*Intervention positively associated with MMF in two trials*	*Mix of positive and null (variation by trial)*
				In the two trials that assessed MMF: infants in SQ‐LNS arm were more likely to receive MMF No differences in MDDS or continued breastfeeding at 18 months. No data reporting for timing of introduction of complementary foods (IYCF data was collected when children were 18 months).	
Byrd et al. (2019)	*Kenya*	*Nutrition, Water and Sanitation RCT* eight‐arm trial with water (W), sanitation (S), handwashing (H) and nutrition (N) interventions. The two nutrition arms received monthly home visits with IYCF counselling + free SQ‐LNS (10 g/2×/day)	*Experimental* 702 clusters, 8246 households eight‐arm cluster RCT: W, S, H, N, Combined WSH; combined WSH + N (Null et al., 2018), active and passive controls	*Intervention not associated with IYCF practices*	*Null*
				Prevalence ratios of intervention groups compared to control presented at Years 1 and 2	
				MDDS: not significant in any group at Years 1 or 2 MMF: not significant in any group at Years 1 or 2 MAD: not significant in any group at Years 1 or 2 No data on timing of introduction of complementary foods	
Kumwenda et al. (2014)	*Malawi*	*RCT of different doses of SQ‐LNS*: Trial not designed to include IYCF BCC; brief nutrition messages conveyed infrequently	*Experimental* Malawi DOSE trial of SQ‐LNS with different zinc doses vs. control (included above in Arimond et al., 2017).	*Intervention not associated w/change in breast milk intake*	*Null*
				Did not report MDD, MMF, MAD or age at introduction of complementary foods There were no significant differences in breastmilk intake in children who received SQ‐LNS vs. controls	
Locks, Dahal, et al. (2019)	*Democratic Republic of Congo*	*Pilot IYCF‐SQ‐LNS program* Intervention included community‐ and facility‐based counselling for mothers on SQ‐LNS, IYCF and handwashing practices and monthly SQ‐LNS distributions for children 6‐12 months	*Quasi‐Experimental* *n* = 2595 Pre and postintervention surveys in representative samples in intervention and control areas	*Intervention positively associated with MMF and age at introduction of solid foods*.	*Positive* *(MMF & age at introduction of complementary foods)* *Null* *(MDD & MAD)*
				Adjusted Prevalence Difference‐in‐difference (DiD) (95% CI):	
				MDD: −0.8 (−4.5, 3.0), *p* = 0.69 Prevalence from Baseline → Endline: Intervention: 6.6% → 2.6% Control: 9.1% → 5.7% MMF: +9.2 (2.7, 15.7), *p* = 0.005 Intervention: 23.9% → 22.2% Control: 30.6% → 23.1% MAD: +1.6 (−0.9, 4.1), *p* = 0.21 Intervention: 2.0% → 0.6% Control: 5.4% → 2.7% Maternal report that solid foods were introduced at 6 months: +54.4 (47.6, 61.1), *p* < 0.001 Intervention: 26.2% → 77.2% Control: 27.7% → 23.7% Additional positive associations reported: breastfeeding within 1 h of birth; introduction of solid foods at 6 months, feeding child in a separate bowl; anemia awareness; owning soap and handwashing behaviors	

Abbreviations: APR, adjusted prevalence ratio; BCC, behavior change communication; DiD, Difference in difference; IYCF, Infant and young child feeding; LNS, lipid‐based nutrient supplement; MAD, minimum acceptable diet; MDD, minimum dietary diversity; MMF, minimum meal frequency; RCT, randomized controlled trial; SQ‐LNS, small quantity lipid‐based nutrient supplement.

### 3.1 Interventions distributing MNP

Seven out of the eight papers on the effect of MNP interventions on IYCF practices reported on the 2008 WHO/UNICEF IYCF complementary feeding indicators of MDD, MMF and/or the MAD (World Health Organization [WHO] & United Nations Children's Fund [UNICEF], 2008), which are derived from a recall of food groups consumed and feeding frequency during the prior 24 h. Given the time frame of the publications, none of the papers used the updated 2021 WHO IYCF indicators (World Health Organization & The United Nations Children's Fund [WHO/UNICEF], 2021). The 2008 indicator for MDD is defined as consuming four or more of the following seven food groups: (1) grains, roots and tubers; (2) legumes and nuts; (3) dairy products; (4) flesh foods; (5) eggs; (6) vitamin‐A‐rich fruits and vegetables; and (7) other fruits and vegetables; home fortificants (such as LNS) do not contribute to dietary diversity calculations. MMF is defined as consuming solid, semisolid or soft foods at least two times per day for infants 6–8 months or at least three times for children 9–23 months who are breastfed or at least four times for nonbreastfed children. MAD is defined as MDD plus MMF. The eighth MNP paper reported on the prevalence of ‘good IYCF practices’, defined as the Infant and Child Feeding Index (ICFI) (Ruel & Menon, 2002) score greater than 6 (Tariqujjaman et al., 2021).

Five of the seven papers from integrated IYCF‐MNP programmes or pilot programmes reported a significant, positive association between the intervention and either MDD, MMF, MAD or the initiation of complementary feeding at 6 months (Table 1). Four articles found that integrated IYCF‐MNP programmes were associated with a significant increase in the probability of children receiving the MDD (Lanou et al., 2019; Locks et al., 2017, 2018; Mirkovic et al., 2016), two studies found a significant, positive association with the MMF (Ford et al., 2021; Mirkovic et al., 2016) and four with MAD (Lanou et al., 2019; Locks et al., 2017, 2018; Mirkovic et al., 2016). Despite significant associations, the prevalence of MDD and MAD remained low in all study areas at endline. The prevalence of MDD in intervention areas in endline surveys ranged from 3.1% in Ethiopia (Geletu et al., 2019) to a maximum of 59.9% in Uganda (Ford et al., 2021). With the exception of Uganda, all other studies reported a prevalence of MDD at endline that was below 50%. The prevalence of MMF at endline was generally higher than MDD, with wide variability from 27.1% in Uganda (Ford et al., 2021) to 91.8% in Burkina Faso (Lanou et al., 2019). All studies reported a MAD at endline below 50% with the highest prevalence (44.8%) observed in Madagascar (Locks et al., 2017). Only three studies used an experimental or quasi‐experimental design (Ford et al., 2021; Lanou et al., 2019; Young et al., 2021); the other studies are from observational studies without a control group, and thus data analyses compare participants in the endline survey to the baseline survey (Locks et al., 2017, 2018) and/or study‐specific ‘compliers’ versus ‘non‐compliers’ from the endline survey for IYCF practices (Locks et al., 2017, 2018; Mirkovic et al., 2016). In the experimental and quasi‐experimental studies, difference‐in‐difference prevalence analyses found no (Young et al., 2021) or modest associations (change in prevalence <10%) between the intervention and IYCF indicators (Ford et al., 2021; Lanou et al., 2019) with the exception of MMF in Uganda (DiD: +18.6% [11.2, 25.9], *p* < 0.0001) (Ford et al., 2021). Furthermore, the significant difference‐in‐differences in the quasi‐experimental studies were partially driven by declines in IYCF indicators in the control areas (Ford et al., 2021; Lanou et al., 2019).

One article reported a decline in IYCF indicators during the period of an IYCF‐MNP programme in Bangladesh (Tariqujjaman et al., 2021). The prevalence of ‘good IYCF practices’—defined as an ICFI > 6 points—was 42.1% at baseline, 45.% at the mid‐point and 31.9% in the endline surveys. In the middle of the evaluation period, the incentive for community‐health workers for IYCF counselling was removed, thus making it impossible to isolate the association between the introduction of MNP with IYCF practices. Notably, the authors reported a significant, positive association between community‐health worker receipt of incentives and IYCF practices, as well as a significant association between effective coverage of MNP and IYCF practices.

Five of the studies reported an indicator of the timing of the introduction of complementary foods. The WHO indicator for the timely introduction of solid, semisolid or soft foods (ISSSF) is defined as the percentage of infants 6–8 months of age who consumed solid, semisolid or soft foods during the previous day. However, given the varying study designs (including cross‐sectional surveys of mothers of older children), some studies provided different indicators for reporting on the age at the introduction of solid foods. Two studies reported the WHO indicator for ISSSF in children 6–8 months (Lanou et al., 2019; Locks et al., 2017), one of which found a statistically significant association between the intervention and the timely introduction of solid foods (Lanou et al., 2019). Three studies used maternal recall on the timing of the introduction of solid foods in the full sample (Ford et al., 2021; Locks et al., 2018; Young et al., 2021), two of which found a statistically significant association between the intervention and the introduction of solid foods at 6 months (Ford et al., 2021; Locks et al., 2018).

### 3.2 Interventions distributing SQ‐LNS

There was greater heterogeneity in the study design and assessment methods in the SQ‐LNS studies compared to the MNP studies. Three of the four papers on SQ‐LNS reported on the effect of SQ‐LNS interventions on the 2008 WHO/UNICEF indicators of MDD, MMF and/or MAD. One study specifically sought to determine whether the distribution of SQ‐LNS displaced breast milk (Kumwenda et al., 2014) and did not include a full panel of IYCF indicators. Kumwenda et al. (2014) used a deuterium oxide dilution method to measure breast milk intake in 9–10‐month‐old children receiving 0, 10, 20 or 40 g of SQ‐LNS per day in Malawi and found no significant difference in intake of breast milk or nonbreast milk water.

Of the three papers that reported on the WHO/UNICEF IYCF indicators, two were from clinical trials of home fortification with SQ‐LNS. One reported on IYCF practices in the WASH Benefits trial, an eight‐arm trial of water, sanitation, handwashing and nutrition interventions in rural Kenya (Byrd et al., 2019). The other study reported on the international lipid‐based nutrient supplements (iLiNS) trials—four related RCTs of SQ‐LNS in sub‐Saharan Africa (Arimond et al., 2017). The WASH Benefits trial found no difference in IYCF indicators at 1 or 2 years of follow‐up in children who received the nutrition intervention (SQ‐LNS + nutrition counselling) compared to children in the ‘active control group' (no SQ‐LNS or nutrition counselling, but monthly home visits with assessment of child's arm circumference) in rural Kenya. By contrast, the iLiNS group reported that the prevalence of children receiving the MMF at 18 months was significantly higher among children randomised to receive SQ‐LNS (compared to no SQ‐LNS) in the two trials that assessed feeding frequency (percentage point differences of 12%–14%, *p* < 0.0001 and *p* = 0.005), even though none of the trials were specifically designed to include an IYCF behaviour change communication (BCC) component (the other two trials did not assess feeding frequency). The iLiNS group did not find significant differences by the treatment group in the prevalence of children consuming the MDD, continued breastfeeding at 18 months or frequent breastfeeding (6 or more times per day) in any of the four trials, but they did report a reduced prevalence of infrequent consumption of animal source foods in the same two trials that found a significant difference in MMF.

Only one study reported on the effect of an integrated IYCF‐SQ‐LNS intervention from a programmatic setting (Locks, Nanama, et al., 2019). The 2‐year pilot intervention was implemented in the Haut‐Katanga District in the Democratic Republic of Congo (DRC) targeting 23,000 pregnant women and infants 0–12 months of age in Kasenga health zone with an enhanced IYCF programme that included monthly SQ‐LNS distribution, as well as resources to support CHW IYCF counselling (trainings, job aids and bicycles for CHWs to travel to remote areas). The study used a quasi‐experimental study design to compare difference‐in‐differences in the intervention and control zones at baseline and endline and found that mothers in the intervention zone were more likely to report the timely introduction of complementary foods, feeding the MMF the previous day, feeding the child in a separate bowl, awareness of anaemia, owning soap and washing hands after defecating and before cooking and feeding the child in the previous day. There were no significant differences in the prevalence of MDD and MAD in the intervention and control zones, where both remained below 10% at both baseline and endline.

## 4 DISCUSSION

This systematic narrative review found that there is limited research on the effect of interventions with home fortification products on IYCF practices. All but one of the studies on interventions providing MNP are from integrated IYCF‐MNP programmes or pilot programmes (there was one pilot programme that did not contain an IYCF component). By contrast, there have been few programmes that have distributed SQ‐LNS, and thus all but one of the published studies are from randomised trials.

Most of the studies from integrated IYCF‐MNP programmes found either no association with IYCF practices or a small beneficial effect on IYCF practices. One study, from Bangladesh, reported a decline in IYCF practices during the implementation of an integrated IYCF‐MNP programme, but important methodological limitations—namely the removal of CHW incentives for IYCF practices in the middle of the programme period—make it difficult to attribute the decline in IYCF practices to the use of home fortification products. From the studies available, it is not possible to isolate whether there is an independent effect on IYCF practices of adding home fortificants to existing, intensive IYCF. In most of the studies, the introduction of MNP was part of an integrated IYCF‐MNP programme, which included the introduction of a new, intensive IYCF BCC component (Ford et al., 2021; Lanou et al., 2019, Locks et al., 2017, 2018; Mirkovic et al., 2016). The only study designed to assess the independent effect of MNP (Young et al., 2021) was conducted in the context of ongoing health and nutrition programmes implemented by CARE, India. The intervention clusters received both MNP and IYCF counselling, while the control group received the same intensity of IYCF counselling as the intervention group. Notably, when the provision of MNP was the only difference between the two intervention groups, there was no difference in the effect on IYCF practices. The provision of MNP did, however, significantly improve children's nutritional status: there was a reduced prevalence of anaemia in the MNP group and a reduced prevalence of stunting among children 12–18 months in the MNP group.

Our findings indicate that in many settings, programme implementers may be capitalising on the introduction of MNP to build more intensive IYCF BCC strategies, which are in turn, improving IYCF practices. The WHO recommends the use of MNP for children 6–23 months in areas with a prevalence of anaemia in young children of 20% or greater, given the strength of the evidence indicating that MNP reduces the risk of anaemia and iron deficiency in young children (WHO, 2016). Our findings support the use of MNP in national and subnational programmes by demonstrating that combined MNP‐IYCF programmes may improve IYCF practices in some settings. Given that MNP programmes often focus on the promotion of MNP, appropriate use and sustained adherence (Pelletier & DePee, 2019), there is the potential for synergy with IYCF counselling that also aims to promote caregiver behaviour change on a daily basis. Harmonised messages could include the following: the introduction of solid foods and MNP at 6 months; the importance of mixing MNP with foods of a thick consistency; and mixing MNP with small portions that children can finish in one setting, while also promoting frequent feedings of small portions of diverse foods and breastfeeding. In addition, the provision of a home fortificant may improve caregiver and/or health worker motivation and thus improve the quantity, quality and uptake of interpersonal counselling and other IYCF BCC (Siekmans et al., 2017).

Despite the association between integrated IYCF‐MNP programmes and improved IYCF practices in several of the studies in this review, the effect sizes were relatively small. MNP fills essential nutrient gaps in the diets of young children; however, improving IYCF, particularly the MDD, will also require interventions that focus on the access and feeding of diverse foods for young children. Furthermore, several of the studies reported a low prevalence of optimal IYCF practices at endline, and several external studies have reported limited MNP coverage and adherence in national and subnational programmes (Locks, Dahal, et al., 2019; Tumilowicz et al., 2019). Taken together, these findings highlight the continued need to strengthen the delivery of community‐based IYCF and MNP programmes (Pelletier & DePee, 2019). Of note, several of the studies in this review reported an association between *effective* programme coverage (defined by each study, usually as receipt of MNP and/or IYCF counselling) and improved IYCF practices (Locks et al., 2017, 2018; Mirkovic et al., 2016; Tariqujjaman et al., 2021), and all of the programmes that found an association between the intervention and IYCF practices included a strong community‐based component (Ford et al., 2021; Lanou et al., 2019; Locks et al., 2017, 2018; Mirkovic et al., 2016). In Nepal, Locks et al. (2018) specifically found that IYCF practices were associated with counselling from Female Community Health Volunteers, but not with counselling from facility‐based health workers. These findings are consistent with research from intensive IYCF counselling programmes (in the absence of home fortification) that have demonstrated that intensive IYCF counselling supplemented with mass media and community mobilisation activities is able to improve IYCF practices compared to standard of care/less‐intensive IYCF counselling (Menon, Nguyen, Saha, Khaled, Kennedy, et al., 2016; Menon, Nguyen, Saha, Khaled, Sanghvi, et al., 2016; Rawat et al., 2017). Given that optimal IYCF practices and the consumption of home fortificants requires daily engagement of the caregiver, consistent, community‐based support for improving both IYCF practices and MNP programme indicators is essential for sustained behaviour change.

In the few studies distributing SQ‐LNS included in this review, we found small or no associations between SQ‐LNS distribution and IYCF practices. This is consistent with qualitative research showing that SQ‐LNS has been well‐accepted in many settings (Adu‐Afarwuah et al., 2011; Lesorogol et al., 2015; Tripp et al., 2011) and that mothers did not report any interference or changes in breastfeeding or complementary feeding practices (Lesorogol et al., 2015). These findings are also supported by the RCT from Honduras which was excluded from this review because the quantity of LNS distributed was larger than standard SQ‐LNS doses (46.3–70 g/day) and was not distributed as a home fortification product (only 30% of mothers reported mixing it with food). Despite the larger quantity of LNS provided to the children in the Honduras study, the 24‐h recall data indicated that the provision of LNS did not affect the quantity of food consumed from any of the food groups assessed, with the exception of increased intake of food groups comprised partially or wholly of the supplement (nuts or nut butters, oils and sweets) (Flax et al., 2015).

In contrast to integrated IYCF‐MNP programmes, there is limited research on the effect of programmes distributing SQ‐LNS on IYCF practices. We found multiple studies from randomised trials assessing whether SQ‐LNS displaces breast milk (Kumwenda et al., 2014) or generally impacts IYCF indicators (Arimond et al., 2017; Byrd et al., 2019), but only one from a programmatic setting (Locks, Nanama, et al., 2019). As of 2020, MNP programmes had been implemented in at least 56 countries worldwide (United Nations Children's Fund [UNICEF], 2022). Recent meta‐analyses have found that SQ‐LNS reduces the risk of stunting and other measures of undernutrition across a range of contexts (Das, Salam, Weise Prinzo, et al., 2019; Dewey, Wessells, et al., 2021; Keats et al., 2021). Given these promising findings, many IYCF programmes may seek to integrate SQ‐LNS; it will be important to continue to assess whether the findings reported in this review are replicable in programmatic settings where resources and key messages are often diluted. In addition, all studies in this review on the effect of SQ‐LNS interventions on IYCF practices are from sub‐Saharan Africa. More research from other geographic regions of the world and from different programmatic contexts—including fragile or food insecure settings—will be important to understand how the integration of SQ‐LNS into nutrition programmes influences IYCF and children's nutrition in different contexts. By contrast, much of the literature on interventions with MNP comes from integrated IYCF‐MNP programmes and pilot programmes; however, several of these studies used observational study designs without control groups and there were no studies from Latin America or the Caribbean. More high‐quality experimental and quasi‐experimental research from programmatic settings using SQ‐LNS and MNP would help determine whether the observed associations are causal, and if so, implementation research examining how, why and which factors are enhancing the causal association could improve integrated IYCF‐home fortification programmatic effectiveness and impact.

This review has strengths and limitations. We systematically reviewed the scientific literature in accordance with the PRISMA guidelines and identified 12 studies assessing the effect of home fortification products on IYCF practices. Given the substantial heterogeneity in the choice of study design (including some without control groups) and selection of IYCF indicators, we were unable to perform quantitative summaries of the research on this topic.

## 5 CONCLUSION

Our findings indicate that programmes that combine home fortification products and IYCF interventions had positive and/or limited influences on IYCF practices. Given that that home fortification products contain several essential nutrients that are lacking in the diets of infants and young children in low‐resource settings, the integration of these products into national and subnational programmes has the potential to improve IYCF practices and nutrition outcomes in vulnerable children (Figure 1).

## AUTHOR CONTRIBUTIONS

Lindsey M. Locks and Maria Elena D. Jefferds developed research questions and research methodology. Lindsey M. Locks and Katharine B. Newell screened abstracts and drafted the first draft of the manuscript. All authors contributed to the manuscript review including the addition of critical intellectual content. All authors read and approved the final version of the manuscript.

## CONFLICT OF INTEREST STATEMENT

The findings and conclusions in this report are those of the authors and do not necessarily represent the official position of the Centres for Disease Control and Prevention or UNICEF. The authors have no conflict of interest to declare.

## Data Availability

Data sharing is not applicable as no new data was generated in this review.
